# Left ventricular systolic function and the pattern of late-gadolinium-enhancement independently and additively predict adverse cardiac events in muscular dystrophy patients

**DOI:** 10.1186/s12968-014-0081-1

**Published:** 2014-09-25

**Authors:** Anca Florian, Anna Ludwig, Markus Engelen, Johannes Waltenberger, Sabine Rösch, Udo Sechtem, Ali Yilmaz

**Affiliations:** Department of Cardiology and Angiology, University Hospital Münster, Albert-Schweitzer-Campus 1, building A1, Münster, 48149 Germany (AY); Division of Cardiology, Robert-Bosch-Krankenhaus, Stuttgart, Germany

**Keywords:** Muscular dystrophy, Cardiomyopathy, CMR, Cardiac events, Prognosis, LGE

## Abstract

**Background:**

Cardiac involvement is a frequent finding in patients with Duchenne (DMD) and Becker (BMD) muscular dystrophies. With this study, we aimed at elucidating the relationship between the phenotypic expression of cardiac involvement and the occurrence of adverse cardiac events in DMD/BMD patients.

**Methods:**

Eighty-eight male DMD/BMD patients (age 29 ± 14 yrs) were prospectively enrolled. All patients underwent cardiovascular magnetic resonance (CMR) comprising cine- and late-gadolinium-enhancement (LGE)-CMR at study entry and were subsequently followed-up for adverse cardiac events. The primary endpoint was defined as all-cause/cardiac death or cardiac transplantation. Secondary endpoints were (1) hospitalization for heart failure and/or (2) occurrence of non-/sustained ventricular tachycardia (VT).

**Results:**

During a mean follow-up time of 47 ± 18 months, the primary endpoint was observed in three (3%) and the secondary endpoint in 21 (24%) patients. On multivariable analysis, LV-EF (HR, 95% CI: 0.94, 0.89-0.97, p = 0.001) and the presence of “transmural” LGE (HR, 95% CI: 2.89, 1.09-7.68, p = 0.033) were the only independent predictors for secondary endpoints. A cut-off for LV-EF of 45% was associated with the highest hazard ratio (HR, 95% CI: 11.50, 4.49-29.43, p < 0.0001) in a Cox regression survival analysis. In the group of patients with a LV-EF (>45%), those patients already showing “transmural” LGE had a significantly lower event-free-survival (HR, 95% CI: 13.48, 1.89-96.12, p = 0.009) compared to those without.

**Conclusions:**

An impaired LV systolic function (LV-EF ≤45%) and a “transmural” pattern of myocardial fibrosis independently predict the occurrence of adverse cardiac events in DMD/BMD patients. Even in DMD/BMD patients with relatively preserved LV-EF (>45%), the simple and visually assessable parameter “transmural LGE” is of additive prognostic value.

## Background

Duchenne and Becker muscular dystrophies (DMD and BMD) are X-linked inherited disorders affecting the synthesis of dystrophin, a large sarcolemmal protein with an essential role in the interaction between cytoskeleton, cell membrane and extracellular matrix [1,2]. The most frequently encountered genetic defects related to these dystrophinopathies are deletions, occurring in 60 to 80% of cases, the remaining being mostly gene duplications or point mutations [3]. The absence of dystrophin protein in DMD and its reduced presence and/or abnormal configuration in BMD lead to skeletal muscle disease with proximal weakness and wasting as well as to progressive cardiomyopathy [2,4]. While in DMD symptoms occur early in childhood and affected patients rarely reach the age of 30 years, BMD is a milder form of dystrophinopathy with later onset in which patients can reach mid to late adulthood [4]. Associations between specific dystrophin mutations and the severity of skeletal involvement as well as onset of cardiomyopathy have been reported [3,5–7].

Since respiratory failure, the former major cause of death in this population, can be better managed by ventilatory support techniques today, cardiac involvement with a non-ischemic, myocarditis-like pattern of left ventricular (LV) myocardial fibrosis leading to non-ischemic dilated cardiomyopathy, progressive heart failure and arrhythmias has become an important cause of morbidity and mortality [1,4,8–10]. In the last years, cardiovascular magnetic resonance (CMR) has been increasingly used for diagnosis as well as for follow-up of cardiac involvement in DMD/BMD patients [11–14]. CMR does not only offer an accurate and reproducible tool for LV systolic function assessment, but also the possibility of non-invasive myocardial tissue characterization and early fibrosis detection based on late gadolinium enhancement (LGE) imaging. Nevertheless, published data on the risk stratification as well as on the prognostic role of CMR in DMD/BMD-related cardiomyopathy are scarce [8].

With this study, we aimed at elucidating the relationship between the phenotype of cardiac involvement (assessed by CMR) and the occurrence of death and adverse cardiac events in DMD/BMD patients.

## Methods

### Study population

Ninety male patients with known muscular dystrophy (MD) were prospectively enrolled in two German centres (Robert-Bosch-Krankenhaus, Stuttgart and Universitätsklinikum Muenster, Muenster) between 2006 and 2013. A diagnosis of muscular dystrophy was previously made in a specialized neurology centre based on skeletal muscle pathology with immunohistochemical dystrophin analyses and/or genetic testing [2]. Eighty-eight of these patients (20 DMD and 68 BMD) underwent a complete CMR study at study inclusion and represented the final study group. The study protocol complies with the Declaration of Helsinki and was approved by the local ethics committee. Informed consent was obtained from the patients prior to study inclusion.

### CMR data acquisition

ECG-gated CMR studies were performed on 1.5-T scanners (Aera, Siemens Medical Solutions, Erlangen, Germany and Achieva, Philips, Best, The Netherlands) using commercially available cardiac software, electrocardiographic triggering, and cardiac-dedicated surface coils. Cine-imaging was performed using a steady-state-free-precession (SSFP) sequence in three long-axis slices (four-, three- and two-chamber) and a stack of short-axis slices completely covering the LV. LGE-imaging was performed using a T1-weighted inversion recovery gradient-echo sequence 10-15 min after intravenous contrast administration (0.15 mmol/kg Magnevist®) in the same imaging planes as the cine-images.

### CMR data analysis

CMR analysis was performed off-line by two experienced readers (AF and AY) blinded to outcomes and clinical characteristics. Ventricular volumes, ejection fraction and LV mass were derived by contouring endo- and epicardial borders on the short-axis cine images and indexed to body surface area. LGE presence and pattern were first visually assessed on the short-and long-axis images by using a model dividing each short–axis slice into 12 sectors and each sector into 3 equal circumferential segments (subendocardial, midmyocardial, subepicardial; in total 36 segments per slice). A LV ejection fraction (EF) less than 55% and/or presence of LGE in at least one myocardial segment were considered signs of cardiac involvement. LGE pattern was globally assessed by two readers (AF and AY) and classified according to its location as: subepicardial, intramural, mixed (subepicardial and intramural). In addition to the three patterns, a fourth transmural pattern was considered whenever all three segments in at least one sector were LGE positive. Eventual discrepancies were resolved by common agreement. Second, LGE extent was planimetered on the short-axis contrast images with the use of ImageJ software (National Institutes of Health, Bethesda, Md, USA) and an image intensity level ≥3 SD above the mean of remote myocardium was used to define LGE indicative of damaged myocardium as described previously and expressed as percentage of total LV mass [15].

### Genetic data analysis

Patients were first categorised as presenting with either deletions, duplications, point mutations or other defects in the dystrophin gene. Thereafter, a subclassification of those patients having dystrophin gene deletions was performed based on previous data relating deletions in specific dystrophin gene domains with the presence and severity of skeletal muscle disease and cardiomyopathy as follows: (1) presence of deletions affecting the amino-terminal domain of dystrophin - known to be associated with DMD/severe skeletal BMD and early onset of cardiomyopathy, (2) presence of deletions affecting exons 45 to 49 preserving Hinge 3 (that encodes a protein sequence responsible for dystrophin flexibility and intrinsic folding) and (3) presence of deletions affecting exons 50 and/or 51 removing or disrupting Hinge 3 [7,16,17].

### Patient follow-up and definition of endpoints

After enrolment and baseline CMR, the patients were followed-up for the occurrence of death and adverse cardiac events until November 2013. Primary endpoints were defined as: (1) all cause death including cardiac death (and particularly sudden cardiac death and death from heart failure) and (2) cardiac transplantation. Secondary endpoints were defined as follows: (1) hospitalization for heart failure and/or (2) non-/sustained ventricular tachycardia (VT) defined as five or more consecutive ventricular beats at a rate of greater than 100/min. In patients with more than one event, the time to the first event was taken into consideration. Follow-up was done by phone calls as well as by periodical (every six months to one year) ambulatory monitoring of potential arrhythmias during a five day period by means of an external event loop recorder (SpiderFlash-t, Sorin Group). This device records electrocardiographic tracings in two different leads during and up to 15min after arrhythmia detection (auto-triggered) and/or patient activation. Subsequently, all ECG recordings were assessed for presence of ventricular arrhythmias. In the case of an event, all explanatory medical records were obtained and reviewed to ensure an appropriate classification.

### Statistical analysis

Continuous variables are expressed as mean ± SD. Skewed variables are expressed as median and interquartile range (IQR). Categorical variables are expressed as frequency with percentage. *t*-Student test was used for comparison of normally distributed characteristics between DMD and BMD patients as well as between patients with and without adverse events. Mann–Whitney U test was used for comparison of non-normally distributed variables. One-way ANOVA with Bonferroni post-hoc correction was used in case of multiple comparisons. Dunnett’s post-hoc was used in case of inequality of variances. The Chi-square test with Yate’s correction was used to compare non-continuous variables expressed as proportions. Pearson correlation (r) was used to assess the relationship between different normally distributed CMR measurements. Intra-observer (AF) and inter-observer (AY) variability for LGE extent was performed in 10 random LGE positive patients and evaluated using Bland-Altman. In order to find independent predictors for the occurrence of a secondary endpoint, a univariable Cox proportional hazards regression analysis was performed first. Second, the parameters with significant p-values were introduced into a Cox regression multivariable analysis. Additionally, a separate model including only three variables: age (the most important clinical variable), LV-EF and LGE characteristics as either (1) dichotomous presence or (2) extent as % of LV mass or (3) pattern was tested in order to avoid the potential for overfitting. The independent predictors thus obtained were used to generate the cumulative event-free survival curves. Statistical analysis was performed using SPSS software for Windows (version 18, SPSS, Chicago Illinois, US). A p-value ≤ 0.05 was considered statistically significant.

## Results

### Patient characteristics

Table 1 summarizes patient characteristics for the total study group (N = 88) as well as according to the muscular dystrophy type, i.e. DMD (N = 20) and BMD (N = 68). Mean age was 29 ± 14yrs (IQR 16 to 41 yrs) and all patients were males. DMD patients (16 ± 5 yrs; IQR 11 to 22 yrs) were significantly younger than BMD ones (33 ± 14 yrs; IQR 23 to 43 yrs; p < 0.001). There was no significant difference in cardiovascular risk factor prevalence between DMD and BMD patients.

**Table 1:** **General patient characteristics**

	**Total (N = 88)**	**DMD (N = 20)**	**BMD (N = 68)**	**p-value**
**Age, yrs**	29 ± 14	16 ± 5	33 ± 14	**< 0.001**
**Male, n (%)**	88 (100)	20 (100)	68 (100)	NA
**NYHA class I-II/III/IV,%**	91 / 9 / 0	100 / 0 / 0	88 / 12 / 0	0.32
**Hypertension, n (%)**	29 (33)	7 (35)	22 (32)	1.00
**Current smoker, n (%)**	10 (11)	1 (5)	9 (13)	0.43
**High cholesterol, n (%)**	2 (2)	0 (0)	2 (3)	1.00
**Diabetes, n (%)**	1 (1)	0 (0)	1 (2)	1.00

DMD – Duchene muscular dystrophy; BMD – Becker muscular dystrophy; NA – non-applicable. Bold numbers indicate significant p-values/parameters.

### CMR findings

The CMR findings are shown in Table 2. Signs of cardiac involvement were observed in 69% (N = 61, 14 DMD and 47 BMD) of the study patients with 51% (N = 45) showing a reduced LV-EF (<55%) and 64% (N = 56) demonstrating presence of LGE. A severely reduced LV-EF (<35%) was found in 10 patients (11%) - all suffering from BMD. Only five patients with negative LGE showed a reduced LV-EF, however, mild in degree (LV-EF >45%) in all of them. BMD patients had significantly higher LV end-diastolic volumes (LV-EDV, p < 0.001), end-systolic volumes (LV-ESV, p < 0.001) and LV mass indexes (p < 0.001) in addition to a significantly lower LV-EF (p = 0.014) compared to DMD patients. Moreover, higher RV end-diastolic (RV-EDV, p < 0.001) as well as end-systolic volume (RV-ESV, p < 0.003) indexes were observed in BMD patients.

**Table 2:** **Overview of CMR findings**

	**Total (N = 88)**	**DMD (N = 20)**	**BMD (N = 68)**	**p-value**
**LV-EDV index, ml/m2**	80 (62–104)	54 (48–64)	85 (69–107)	**<0.001**
**LV-ESV index, ml/m2**	33 (24–56)	21 (14–30)	38 (27–62)	**<0.001**
**LV mass index, mg/m2**	58 ± 16	42 ± 14	63 ± 14	**<0.001**
**LV-EF,%**	53 ± 14	60 ± 11	51 ± 15	**0.014**
**LV-EF <55%, n (%)**	45 (51)	7 (35)	38 (56)	0.13
**RV-EDV index, ml/m2**	63 (51–78)	49 (40–59)	65 (58–81)	**<0.001**
**RV-ESV index, ml/m2**	30 (23–36)	24 (19–29)	31 (25–39)	**0.003**
**RV-EF,%**	53 ± 10	51 ± 9	53 ± 10	0.32
**LGE presence, n (%)**	56 (64)	13 (65)	43 (63)	1.00
**LGE extent,%**	16 ± 13 (n = 56)	13 ± 12 (n = 13)	17 ± 14 (n = 43)	0.37
**Transmural LGE, n (%)**	20 (23)	5 (25)	15 (22)	0.77

DMD – Duchene muscular dystrophy; BMD – Becker muscular dystrophy; LV – left ventricle; RV – right ventricle; EDV – end diastolic volume; ESV – end systolic volume; EF – ejection fraction; LGE – late gadolinium enhancement. Bold numbers indicate significant p-values/parameters.

An exclusively non-ischemic pattern of LGE was detected in all LGE-positive patients (N = 56) showing the following LGE distribution: 34% (N = 19) subepicardial, 7% (N = 4) intramural, 23% (N = 13) mixed (intramural and subepicardial) and 36% (N = 20) transmural. Presence of LGE in the LV lateral free wall was observed in 96% (N = 54), while septal, inferior and anterior wall involvement was present in 55% (N = 31), 50% (N = 28) and 34% (N = 19), respectively.

A good reproducibility was obtained for LGE extent measurements both for intra- (−4.8 to 3.8%, 95% confidence interval) and for inter-observer variability (−7.5 to 3.9%, 95% confidence interval).

**Figure 1:**
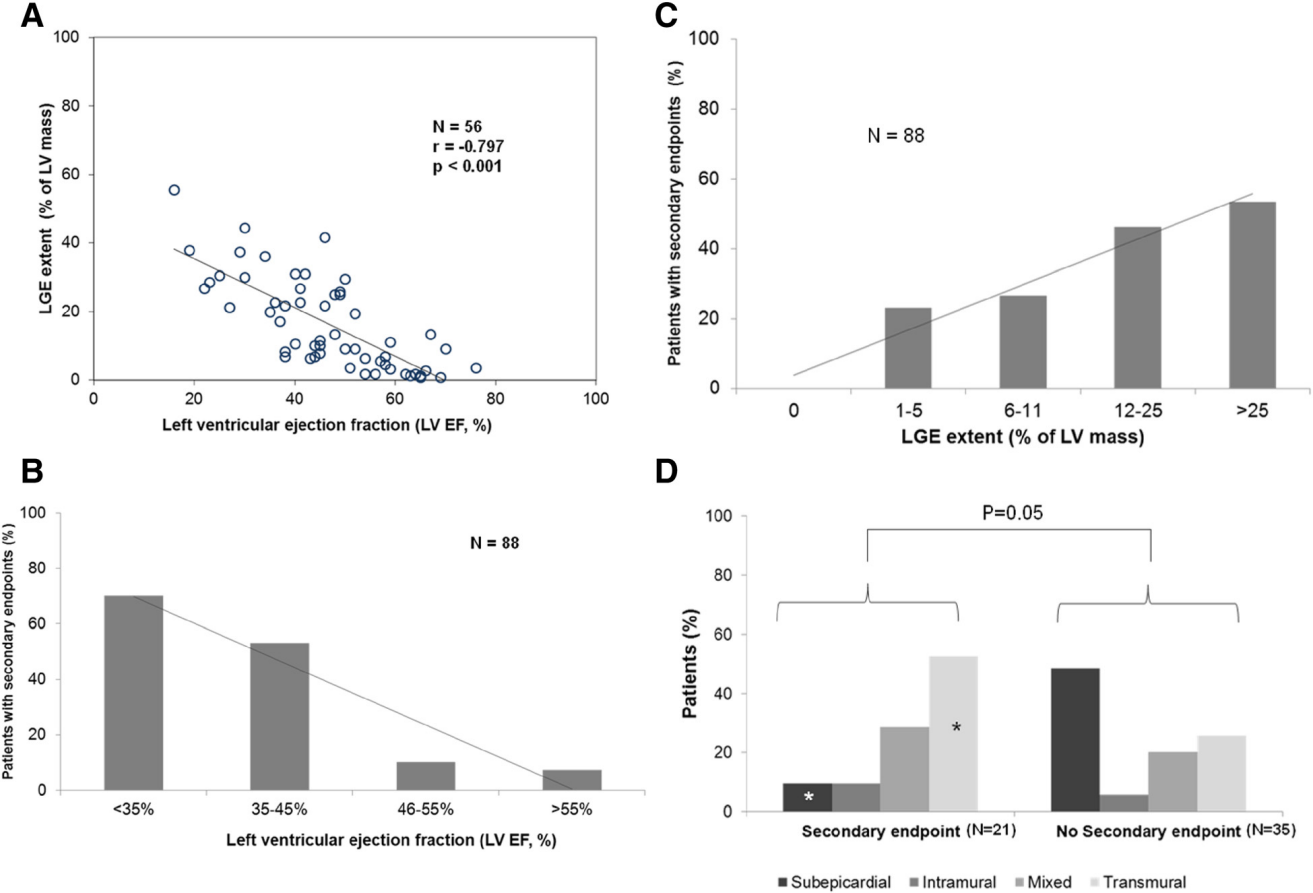
**Distribution of LV-EF, LGE and cardiac events.** **(A)** Scatter plot showing the high correlation between left ventricular ejection fraction (LV-EF) and late-gadolinium-enhancement (LGE) extent. **(A)** Scatter plot showing the high correlation between left ventricular ejection fraction (LV-EF) and late-gadolinium-enhancement (LGE) extent. (B) and (C) Distribution of secondary endpoints according to the degree of LV systolic impairment **(B)** and to the extent of LGE **(C)**. For both parameters LV-EF and LGE extent a linear relationship was detected between the decrease in LV-EF / increase in LGE extent and the frequency of secondary endpoints. **(D)** The distribution of different LGE patterns among patients with and without secondary endpoints. Isolated subepicardial LGE was more frequently seen in patients without events while transmural LGE was a common finding in patients with events. (*- p < 0.10 vs. similar pattern subtype in the other group).

A high correlation was found between LV-EF and LGE extent (Pearson r = −0.797, p < 0.001; Figure 1A). Moreover, there was a significant relationship between the pattern of LGE and the degree of LV systolic dysfunction: 90% of the patients with transmural LGE and 100% of the patients with mixed LGE demonstrated a LV-EF <55% in comparison to only 46% of patients with isolated subepicardial LGE (p = 0.001). There was no significant difference between DMD and BMD regarding LGE prevalence (p = 1.00) and extent (p = 0.37). However, there was a significant difference in the distribution of LGE pattern between BMD and DMD, the latter patients presenting more frequently with isolated subepicardial LGE (p = 0.037).

### Occurrence and distribution of endpoints

As shown in Table 3, during a mean total follow-up of 47 ± 18mo (IQR 37 to 57 mo) three primary endpoints occurred: two deaths (of which one was cardiac) and one heart transplantation. Additionally, 22 secondary endpoints were observed in 21 patients (one patient experienced occurrence of two secondary endpoints at the same time): 8 heart failure hospitalizations, 1 sustained VT episode and 13 non-sustained VT episodes. Thus, a secondary endpoint occurred in 24% (N = 21) of patients at a mean time to event of 28 ± 18 mo (IQR 15 to 41 mo) since study inclusion.

**Table 3:** **Distribution of primary and secondary endpoints**

	**Total (N = 88)**	**DMD (N = 20)**	**BMD(N = 68)**	**p-value**
**Mean follow-up duration, months**	47 ± 18	47 ± 13	47 ± 19	0.92
**Primary endpoint, n (%)**	3 (3)	1 (5)	2 (3)	0.54
**Secondary endpoint, n (%)**	21 (24)	1 (5)	20 (29)	**0.034**
**Hospitalization for HF, n (%)**	8 (9)	0 (0)	8 (12)	0.190
**Non-/sustained VT, n (%)**	14 (16)	1 (5)	13 (19)	0.175

DMD – Duchene muscular dystrophy; BMD – Becker muscular dystrophy. HF – heart failure; VT – ventricular tachycardia. Bold numbers indicate significant p-values/parameters.

### Relationship between CMR findings and secondary endpoint occurrence

Table 4 shows CMR findings in patients with (N = 21) and without (N = 67) a secondary endpoint. Patients with secondary endpoints had significantly higher LV-EDV (p < 0.001), LV-ESV (p < 0.001), LV mass indexes (p < 0.001) and significantly lower LV-EF (p < 0.001) than patients without a secondary endpoint (Figure 1B). In addition, LGE presence (p < 0.001) and extent (p < 0.001) were significantly higher in patients with a secondary endpoint (Figure 1C). Moreover, isolated subepicardial LGE was more frequently seen in patients without events whereas a transmural pattern of LGE was a common finding in patients with a secondary endpoint (Figure 1D).

**Table 4:** **CMR parameters in relation to secondary endpoint occurrence**

	**Total (N = 88)**	**Secondary endpoint (N = 21)**	**No endpoint (N = 67)**	**p-value**
**Age, yrs**	29 ± 14	39 ± 11	27 ± 14	**0.001**
**DMD/BMD, n (%)**	20 (23) / 68 (77)	1 (5) / 20 (95)	19 (28) / 48 (72)	**0.034**
ACEi/ARB, n (%)*	62 (71)	20 (95)	42 (63)	**0.005**
Beta-blocker, n (%)*	36 (41)	16 (76)	20 (30)	**< 0.001**
Aldosterone antagonist, n (%)*	9 (10)	7 (33)	2 (3)	**< 0.001**
Corticosteroid, n (%)*	6 (7)	0 (0)	6 (9)	0.329
**Time to event, months**	28 ± 18	28 ± 18	-	-
**LV-EDV index, ml/m2**	80 (62–104)	101 (84–117)	73 (54–93)	**< 0.001**
**LV-ESV index, ml/m2**	33 (24–56)	63 (47–82)	29 (20–43)	**< 0.001**
**LV mass index, mg/m2**	58 ± 16	71 ± 12	54 ± 16	**< 0.001**
**LV-EF,%**	53 ± 14	39 ± 12	57 ± 13	**< 0.001**
**RV-EDV index, ml/m** ^**2**^	63 (51–78)	61 (56–78)	63 (49–78)	0.29
**RV-ESV index, ml/m** ^**2**^	30 (21–36)	30 (24–40)	30 (22–35)	0.13
**RV-EF,%**	53 ± 10	51 ± 12	53 ± 9	0.54
**LGE presence, n (%)**	56 (64)	21 (100)	35 (52)	**< 0.001**
**LGE extent,%**	16 ± 13 (n = 56)	20 ± 12 (n = 21)	8 ± 12 (n = 35)	**< 0.001**
**Transmural LGE, n (%)**	20 (23)	11 (52)	9 (13)	**0.001**

*- at last follow-up.

DMD – Duchene muscular dystrophy; BMD – Becker muscular dystrophy; NA – non-applicable; LV – left ventricle; RV – right ventricle; EDV – end diastolic volume; ESV – end systolic volume; EF – ejection fraction; LGE – late gadolinium enhancement. Bold numbers indicate significant p-values/parameters.

### Predictors of adverse events

The results of the univariable regression analyses regarding predictors for secondary endpoints are shown in Table 5. Apart from functional parameters such as LV-EDV, LV-ESV, LV mass index as well as LV-EF, structural parameters comprising (categorical) presence of any LGE, LGE extent and presence of a transmural LGE pattern were significantly associated with the occurrence of secondary endpoints.

**Table 5:** **Predictors for secondary endpoint occurrence in the study group (N = 88)**

	**Univariable analysis**	
	**HR (95% CI)**	**p-value**
**Age**	1.05 (1.02-1.09)	**0.001**
**BMD**	6.76 (0.91-50.45)	0.06
**LV-EDV index, ml/m2**	1.03 (1.02-1.04)	**< 0.001**
**LV-ESV index, ml/m2**	1.03 (1.02-1.05)	**< 0.001**
**LV mass index, mg/m2**	1.07 (1.03-1.10)	**< 0.001**
**LV-EF,%**	0.91 (0.88-0.94)	**< 0.001**
**RV-EDV index, ml/m** ^**2**^	1.00 (0.99-1.03)	0.34
**RV-EF,%**	0.98 (0.94-1.03)	0.48
**LGE presence**	46.26 (1.23-1736.43)	**0.038**
**LGE extent,%**	1.07 (1.04-1.11)	**< 0.001**
**Transmural LGE**	6.96 (2.83-17.12)	**< 0.001**

BMD – Becker muscular dystrophy; LV – left ventricle; RV – right ventricle; EDV – end diastolic volume; ESV – end systolic volume; EF – ejection fraction; LGE – late gadolinium enhancement. Bold numbers indicate significant p-values/parameters.

In the first Cox regression multivariable model that included all variables associated with events in the preceding univariable analysis, no independent predictors were obtained. Considering the limited number of events, a separate model including only three variables (1: age, 2: LV-EF and 3: one of the LGE characteristics comprising presence, extent or pattern) was additionally performed. In this model, LV-EF was the strongest independent predictor (HR, 95% CI: 0.94, 0.89-0.97, p = 0.001) of adverse events. While a transmural pattern of LGE (HR, 95% CI: 2.89, 1.09-7.68, p = 0.033) was also an independent predictor for secondary endpoints, neither the categorical presence of LGE per se (p = 0.93) nor the extent of LGE (p = 0.70) were independently associated with adverse events. Several cut-offs for LGE extent (>2%, >5%, >10% and >15%, respectively) were also included in the multivariable model (that was limited to three variables) along with age and LV-EF. None of these cut-offs was independently associated with secondary endpoint occurrence.

**Figure 2:**
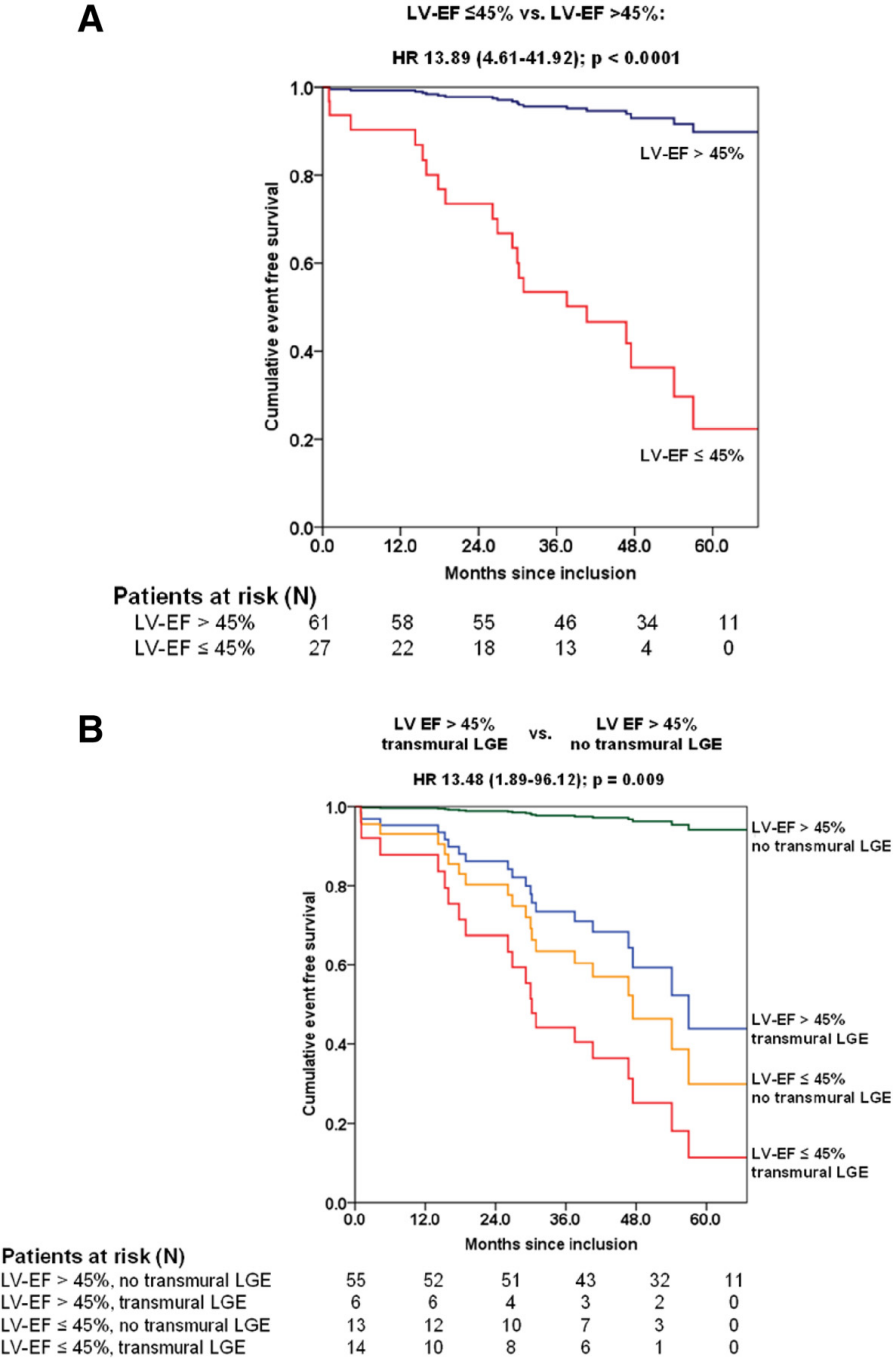
**Cox regression survival curves for secondary endpoint occurrence according to (A) a cut-off for left ventricular ejection fraction (LV-EF) of 45% and (B) both a cut-off for LV-EF of 45% and the presence/absence of transmural late-gadolinium-enhancement (LGE).** Among patients with a LV-EF >45%, those showing transmural LGE had a lower event free survival in comparison to patients without transmural LGE.

A cut-off for LV-EF of 45% was associated with the highest hazard ratio in the Cox regression survival analysis for secondary endpoints (Figure 2A). Moreover, as shown in Figure 2B, among patients with a LV-EF >45%, those showing transmural LGE in at least one myocardial segment had a significantly lower event free survival (HR, 95% CI: 13.48, 1.89-96.12, p = 0.009) compared to those without transmural LGE. Among patients with a LV-EF ≤ 45%, there was no significant difference in event free survival between those with and without transmural LGE (Figure 2B).

### Genetic analysis findings

Results of a genetic analysis were available in 92% (N = 81) of patients. Table 6 summarizes the prevalence of different dystrophin gene mutation types. Deletions were the most frequently encountered genetic defects (70%, N = 56). DMD patients showed significantly more deletions affecting exons 50 and/or 51 (affecting Hinge 3) while BMD patients presented significantly more deletions affecting exons 45–49 (preserving Hinge 3).

**Table 6:** **Distribution of dystrophin gene mutation types in the study population**

**Dystrophin gene mutation type**	**Total (N = 81)**	**DMD (N = 20)**	**BMD (N = 61)**	**p-value**
**Deletions affecting the amino-terminal domain, n (%)**	11 (14)	1 (5)	10 (16)	0.28
**Deletions affecting exons 45–49, n (%)**	30 (37)	1 (5)	29 (48)	**<0.001**
**Deletions affecting exons 50 and/or 51, n (%)**	15 (19)	8 (40)	7 (12)	**0.008**
**Duplication, n (%)**	11 (14)	4 (20)	7 (12)	0.45
**Point mutation, n (%)**	4 (5)	2 (10)	2 (3)	0.25
**Other, n (%)**	10 (12)	4 (20)	6 (10)	0.25

DMD – Duchene muscular dystrophy; BMD – Becker muscular dystrophy. Bold numbers indicate significant p-values/parameters.

No significant relationship between MD type and secondary endpoints was found in the respective regression analysis, although a significantly larger percentage of BMD patients experienced adverse events as compared to DMD. Keeping the limited number of patients in each subgroup in mind, no significant difference in prevalence of different genetic defects in relation to the occurrence of adverse events was found (Table 7).

**Table 7:** **Distribution of dystrophin gene mutation types according to secondary endpoint occurrence**

**Dystrophin gene mutation type**	**Total (N = 81)**	**Secondary endpoint (N = 18)**	**No endpoint (N = 63)**	**p-value**
**Deletions affecting the amino-terminal domain, n (%)**	11 (14)	3 (17)	8 (13)	1.00
**Deletions affecting exons 45–49, n (%)**	30 (37)	7 (39)	23 (37)	0.79
**Deletions affecting exons 50 and/or 51, n (%)**	15 (19)	2 (11)	13 (21)	0.75
**Duplication, n (%)**	11 (14)	5 (28)	6 (10)	0.13
**Point mutation, n (%)**	4 (5)	1 (5)	3 (5)	1.00
**Other, n (%)**	10 (12)	0 (0)	10 (16)	0.06

DMD – Duchene muscular dystrophy; BMD – Becker muscular dystrophy.

Regarding the interaction between the underlying genotype and the cardiac phenotype (as assessed by CMR), no significant differences in LV functional parameters, i.e. volume indexes (p = 0.16 for LV-EDV and p = 0.19 for LV-ESV index, respectively) or LV-EF (p = 0.39) were observed among the respective groups with different types of dystrophin gene mutations.

**Figure 3:**
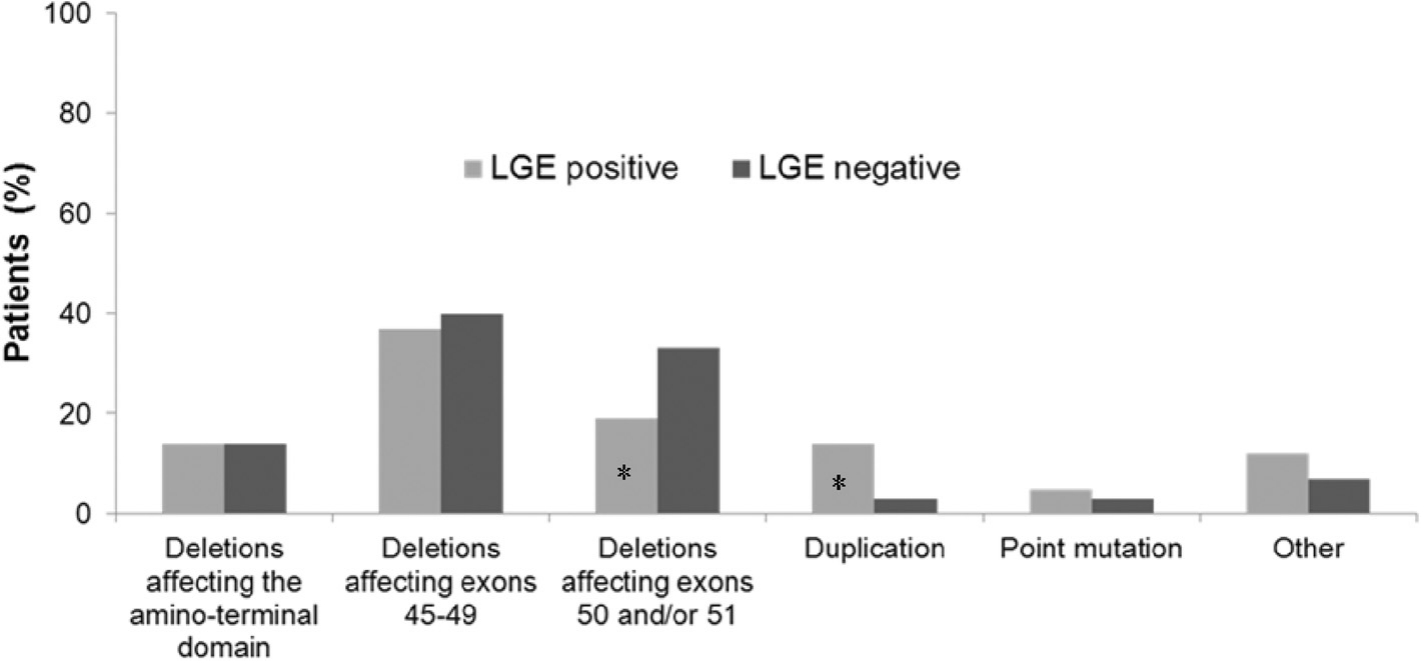
The distribution of different dystrophin gene mutation types among patients with and without positive late-gadolinium-enhancement (LGE) (* - p < 0.05 vs. similar defect in the other group).

Interestingly, patients with dystrophin deletions affecting exons 50 and/or 51 demonstrated more frequently absence of LGE (67%, N = 10, p = 0.015). In contrast, patients with dystrophin duplications showed more frequently presence of LGE (91% N = 10, p = 0.047) (Figure 3). Moreover, mean LGE extent was significantly higher in patients with dystrophin gene duplications compared to those with dystrophin deletions affecting exons 50 and/or 51 (3 ± 5% vs. 17 ± 12%, post-hoc p = 0.040). There was no significant difference in the LGE pattern among the different types of dystrophin gene defects (p = 0.70).

**Figure 4:**
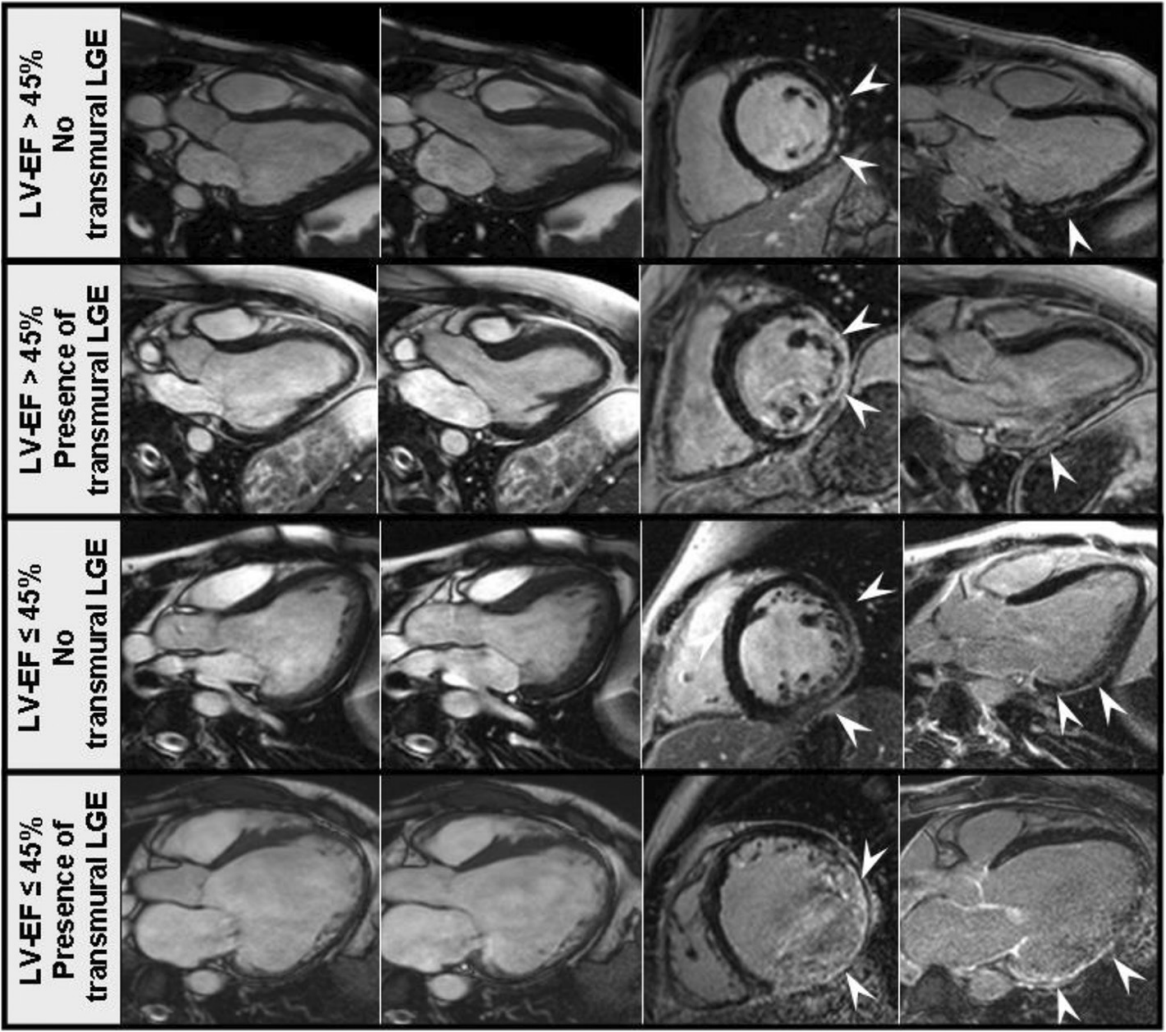
**Examples of cine-CMR images in the 3-chamber-view (end-diastolic and end-systolic frames, left panels) and LGE-CMR images in midventricular short-axis and 3-chamber-views (right panels) from four muscular dystrophy patients with different stages of cardiomyopathy.** In the upper panel, images are from a 22-year-old Becker muscular dystrophy (BMD) patient with subepicardial LGE as only sign of cardiac involvement. The images in the second panel are from a 27-year-old patient with Duchenne muscular dystrophy (DMD) with non-sustained ventricular tachycardia (VT) episodes showing presence of transmural LGE, however, only a mildly impaired left ventricular systolic function. The images in the third panel are from a 25-year old BMD patient showing an impaired LV-EF and only subepicardial LGE that had a heart failure hospitalization. In the lowest panel, images are from a 35-year old BMD patient with an already established picture of dilated cardiomyopathy, a high-grade left ventricular systolic dysfunction and a transmural pattern of LGE who did undergo heart transplantation.

## Discussion

To the best of our knowledge, this is the first follow-up study including DMD and BMD patients that aimed to evaluate the cardiac phenotype (based on CMR studies) in relation to the underlying genotype and to identify independent predictors for adverse cardiac events in a mid- to long-term follow-up period of approximately four years. We could show that the degree of LV systolic dysfunction bears the strongest (independent) predictive value for adverse cardiac events in DMD/BMD patients. Moreover, the addition of the simple, visually assessable CMR parameter “presence of transmural LGE” showed important independent as well as additive value in the further risk stratification of DMD/BMD patients.

### Cardiac involvement in muscular dystrophy patients as assessed by CMR

The current study findings are in accordance with previously published reports, revealing cardiac involvement as defined by an impaired LV-EF and/or the presence of LGE in approximately 70% of DMD/BMD patients [1,4,18]. Similarly to what has been previously shown, occurrence of LGE - with a predominance in the subepicardium of the LV lateral free wall - preceded LV systolic dysfunction in the majority of the patients. Thus, LGE-CMR was shown once again to be a sensitive tool for early detection of cardiac involvement in MD – when global LV function is not yet affected. Moreover, the degree of LV dysfunction was related to the total extent of LGE: patients with advanced cardiomyopathy more frequently showed occurrence of mixed (intramural septal in addition to subepicardial LV lateral wall) LGE as well as presence of a (regional) transmural pattern of LGE (Figure 4) [2,19].

The frequency of cardiac disease was similar for both DMD and BMD patients in our study group. However, BMD patients demonstrated a more advanced stage of cardiac disease compared to the DMD group. This finding is easily explained by the age-dependent occurrence and progression of cardiac disease in DMD/BMD and by the fact that DMD patients were significantly younger [4,20]. The predominance of isolated LGE in the subepicardium of the LV lateral free wall in DMD patients further confirms the early nature of this pattern in the course of cardiomyopathy development whereas additional septal LGE and/or transmural LGE in the LV lateral free wall mostly occurs in advanced stages [19].

### Occurrence of endpoints and risk stratification for adverse events

During the median 4-year follow-up period, only 3% (N = 3) of the study patients had a primary endpoint (two deaths and one heart transplantation) and 24% (N = 21) experienced secondary endpoints (heart failure hospitalizations and/or sustained/non-sustained VT episodes). Due to the limited number of primary endpoints, a meaningful correlation analysis in order to identify predictors for adverse primary events was not possible. However, the occurrence of a secondary endpoint was associated with older age and advanced cardiac disease that was assessed by both functional (cine-CMR) and structural parameters (LGE characteristics including LGE presence, extent and pattern).

Not surprisingly, the functional parameter “LV-EF” proved to be the strongest independent predictor for adverse cardiac events – followed by the parameter “transmural pattern of LGE”. Recently, Diegoli et al. studied patients with genetically proven dystrophin gene defects and already established DCM and reported an event rate of 50% during a median follow-up of five years (comprising nine deaths and eight heart transplantations) [8]. Obviously, the patients in the study of Diegoli et al. suffered from more advanced cardiomyopathy with established DCM and a mean LV-EF of 30 ± 11% only compared to a mean LV-EF of 53 ± 14% in our study group. In contrast, Schram et al. studied a relatively young DMD patient group (9 ± 4 yrs) with normal or only mildly impaired LV-EF at baseline and evaluated the role of prophylactic steroid therapy on mortality and cardiovascular outcomes. The authors reported no deaths in the first five years of follow-up. Moreover, 96% of their patients demonstrated a preserved LV-EF of ≥45% at 5-year follow-up [21]. Taken together, these data clearly illustrate the (age-dependent) association between the degree of LV systolic dysfunction and the occurrence of adverse cardiac events in MD patients.

### A new prognostic indicator: presence of “transmural LGE”

Whereas the association of a reduced LV-EF with the occurrence of adverse cardiac events was shown for ischemic as well as different non-ischemic cardiomyopathies, the unique finding of the present study was obtained when LGE characteristics (including LGE presence, extent and pattern) were carefully looked at: A second independent predictor for adverse cardiac events was the presence of a transmural pattern of LGE (assessed as a dichotomous parameter) - but not the categorical presence of LGE per se or the extent of LGE. At first view, this finding seems to be in disagreement with previously established data in other non-ischemic cardiomyopathies, e.g. DCM, myocarditis or hypertrophic cardiomyopathy; in these forms of cardiac disease the qualitative presence of any LGE (without further quantification or classification) was suggested to be of prognostic value [22–25]. However, a possible explanation for our different finding may be due to the different timing of focal myocardial fibrosis occurrence (depicted by LGE-CMR in the disease course) in different forms of non-ischemic cardiomyopathies. While in our DMD/BMD study group presence of LGE was depicted in 64% of cases and preceded LV-EF decline, e.g. Gulati et al. showed presence of intramural LGE in (only) 30% of DCM patients [26]. Moreover, those LGE-positive DCM patients showed significantly more severe LV adverse remodelling than DCM patients without LGE suggesting that LV dysfunction supposedly precedes occurrence of LGE in (idiopathic) DCM [26].

Furthermore, when looking at biopsy proven viral myocarditis, patients frequently show subepicardial or intramural patterns of LGE (similar to DMD/BMD ones) and presence of LGE was suggested to be the best independent predictor of cardiac mortality [22,27,28]. However, in case of (viral) myocarditis, a subepicardial pattern of LGE is frequently observed in the LV free wall in the acute phase with a shrinkage of LGE (or sometimes even a complete disappearance) in the further disease course that is explained by resolution of myocardial inflammation. Hence, timing of CMR is critical in order to depict the total extent of myocardial inflammation/damage in the acute phase. Moreover, LGE in DMD/BMD patients is caused by progressive myocardial fibrosis as a consequence of ongoing cardiomyocyte cell death due to dystrophin-deficiency. Consequently, naturally occurring LGE does not disappear in DMD/BMD patients and is rather extending in the further disease course with a continuous shift in the pattern from subepicardial to transmural.

Moreover, in a recent publication from our group, we have already shown the diagnostic value of novel myocardial tissue characterization techniques such as the measurement of extracellular volume fraction (ECV) based on T1-mapping pre- and post-contrast agent administration in BMD patients [29]. We could demonstrate that subtle diffuse myocardial fibrosis is present in LGE-negative myocardial areas. Hence, ECV measurement may also have a future role in risk stratifying of MD patients.

The present study findings underline the importance of looking beyond the simple presence of LGE. In addition to that, the pattern of occurrence and further progression of LGE have to be considered, since the pattern and development of LGE reflect different pathomechanisms in different disease forms, e.g. in genetically-driven versus acquired cardiac diseases: increased cell fragility and subsequent cell death with fibro-fatty myocardial replacement fibrosis in DMD/BMD vs. an inflammatory mediated process in myocarditis [30].

### Additive prognostic value of functional and structural CMR

In the present study, a LV-EF cut-off value of 45% resulted in the highest hazard ratio for identifying /discriminating patients with adverse cardiac events. Importantly, when we looked at those patients with a LV-EF >45% (who were classified as low-risk according to functional assessment only), the additive depiction of a transmural pattern of LGE (in at least one myocardial segment) allowed to identify those patients in this subgroup with significantly increased risk for adverse cardiac events - despite a LV-EF >45%. We hypothesize that (slow and rather diffuse) LGE progression to new myocardial regions directly relates to the continuous decline in LV systolic function, while the regional occurrence of transmural LGE - despite preserved LV systolic function - bears an independent negative prognostic value already in the early stages of cardiomyopathy from a functional point of view. Obviously, the identification of DMD/BMD patients who are at increased risk for adverse cardiac events despite a preserved LV systolic function (LV-EF >45%) based on the detection of transmural LGE during CMR is not possible when cardiac work-up in these patients is limited to conventional echocardiography.

### Relationship between genotype and cardiac phenotype

In the present study, DMD/BMD patients having dystrophin gene deletions in exons 50 and/or 51 showed less LGE, while patients with dystrophin gene duplications demonstrated more LGE regarding both LGE prevalence and extent. However, no association between the underlying genotype and the severity of cardiomyopathy, i.e. the degree of LV systolic impairment or LV dilatation, was found. Several previous studies attempted to relate the underlying dystrophin gene defect to the cardiac phenotype [5,31]. For example, Kaspar et al. related the locus of dystrophin gene mutation to the timing of cardiomyopathy onset, with an early onset for deletions affecting the amino-terminal domain (exons 2 to 9) and a later onset for deletions removing part of the central rod domain and hinge 3 (exons 50 and/or 51) [6]. In principle, our findings are not conflicting with the above data from Kaspar et al. However, one has to consider that there were differences in the study group (Kaspar et al. included patients with BMD and X-linked dilated cardiomyopathy) and did not have comprehensive CMR data available [6].

Moreover, considering our preliminary observations that a) the severity of cardiomyopathy may tremendously differ between (unrelated) MD patients of the same age having exactly the same dystrophin gene deletion and b) a similar severity of cardiac disease is frequently detected in MD siblings of similar age (suffering from the same dystrophin gene mutation), the presence of additional genetic modifiers that determine cardiac disease severity is highly expected. Hence, future comprehensive genetic analyses such as whole-exome sequencing or comprehensive epigenetic analyses (OMICS) in MD patients may help to identify such cardiac disease-modifying elements/pathways. However, non-genetic factors such as preserved physical activity (with consecutive cardiac mechanical stress) - particularly in BMD patients with no or only minor skeletal myopathy - may also play an important role regarding the occurrence and severity of cardiomyopathy in these patients.

### Clinical implications of the present results

Taken together, our current findings indicate that, in addition to its already acknowledged role in the early diagnosis of cardiac involvement in DMD/BMD patients, LGE-CMR plays also a prognostic role in risk stratification for adverse cardiac events and might also be of potential therapeutic value [1]. For example, the detection of a transmural pattern of LGE in a DMD/BMD patient with a normal or only mildly reduced LV-EF could represent an appropriate time-point for starting anti-arrhythmic therapy with ß-blockers as well as for implementation of ACE inhibitors or even steroid therapy that were proven to have at least some delaying effects on negative cardiac remodelling [5,14,21,32]. In addition, considering the overall prevalence of ventricular arrhythmias in our study group of 16% (VT/nsVT episodes), we suggest that an ambulatory arrhythmia monitoring should be considered in all patients with cardiac involvement regardless of LV-EF and with a more intensive follow-up frequency in those patients who are at increased risk, e.g. those with transmural LGE and/or a LV-EF ≤ 45%.

### Study limitations

A first limitation of the current study is the limited number of deaths/heart transplantations which prevented us to perform a multivariable risk analysis for primary endpoints. In the light of the above discussion, this is primarily due to the inclusion of relatively young patients with rather preserved cardiac function (only 11% with severe LV systolic dysfunction). Second, no comprehensive and meaningful genotype-associated prognosis assessment could be presently made. Obviously, the main reason was the discrepancy between the large number of different genetic mutations and the limited number of secondary endpoints that narrow the possibility for a robust subgroup analysis. Third, no complete data regarding serum natriuretic peptides were available for analysis. These biomarkers have an established prognostic role in heart failure patients and should be also addressed in future studies [33].

Finally, no myocardial strain data were available for the current analysis. Myocardial strain abnormalities have been shown to occur early in the disease course in DMD patients while LV-EF is still preserved and may have a role in the risk stratification of patients with LV-EF > 45% [34].

## Conclusions

Cardiac involvement is a frequent finding in DMD/BMD patients and can be accurately detected in its early stages by comprehensive CMR studies. An impaired LV systolic function (LV-EF ≤45%) and a “transmural” pattern of myocardial fibrosis independently predict the occurrence of adverse cardiac events in DMD/BMD patients. Even in DMD/BMD patients with relatively preserved LV-EF (>45%), the simple and visually assessable parameter “transmural LGE” is of additive prognostic value.

## Abbreviations

BMD: Becker muscular dystrophy
CMR: Cardiovascular magnetic resonance
DCM: Dilative cardiomyopathy
DMD: Duchenne muscular dystrophy
LGE: Late-gadolinium-enhancement
LV: Left ventricle
LV-EDV: Left ventricular end-diastolic volume
LV-ESV: Left ventricular end-systolic volume
LV-EF: Left ventricular ejection fraction
MD: Muscular dystrophy
nsVT: Non-sustained ventricular tachycardia
RV: Right ventricle
RV-EDV: Right ventricular end-diastolic volume
SSFP: Steady-state-free-precession
VT: Ventricular tachycardia
